# Experiments testing macroscopic quantum superpositions must be slow

**DOI:** 10.1038/srep22777

**Published:** 2016-03-09

**Authors:** Andrea Mari, Giacomo De Palma, Vittorio Giovannetti

**Affiliations:** 1 NEST, Scuola Normale Superiore and Istituto Nanoscienze-CNR, I-56126 Pisa, Italy; 2 INFN, Edificio C, Largo Bruno Pontecorvo, 3, 56127 Pisa PI, Italy

## Abstract

We consider a thought experiment where the preparation of a macroscopically massive or charged particle in a quantum superposition and the associated dynamics of a distant test particle apparently allow for superluminal communication. We give a solution to the paradox which is based on the following fundamental principle: any local experiment, discriminating a coherent superposition from an incoherent statistical mixture, necessarily requires a minimum time proportional to the mass (or charge) of the system. For a charged particle, we consider two examples of such experiments, and show that they are both consistent with the previous limitation. In the first, the measurement requires to accelerate the charge, that can entangle with the emitted photons. In the second, the limitation can be ascribed to the quantum vacuum fluctuations of the electromagnetic field. On the other hand, when applied to massive particles our result provides an indirect evidence for the existence of gravitational vacuum fluctuations and for the possibility of entangling a particle with quantum gravitational radiation.

The existence of coherent superpositions is a fundamental postulate of quantum mechanics but, apparently, implies very counterintuitive consequences when extended to macroscopic systems. This problem, already pointed out since the beginning of quantum theory through the famous Schrödinger cat paradox^1^, has been the subject of a large scientific debate which is still open and very active.

Nowadays there is no doubt about the existence of quantum superpositions. Indeed this effect has been demonstrated in a number of experiments involving microscopic systems (photons^2^,^3^, electrons^4^,^5^, neutrons^6^, atoms^7^,^8^, molecules^9^,^10^, *etc.*). However, at least in principle, the standard theory of quantum mechanics is valid at any scale and does not put any limit on the size of the system: if you can delocalize a molecule then nothing should forbid you to delocalize a cat, apart from technical difficulties. Such difficulties are usually associated with the impossibility of isolating the system from its environment, because it is well known that any weak interaction changing the state of the environment is sufficient to destroy the initial coherence of the system.

In this work we are interested in the ideal situation in which we have a macroscopic mass or a macroscopic charge perfectly isolated from the environment and prepared in a quantum superposition of two spatially separated states. Without using any speculative theory of quantum gravity or sophisticated tools of quantum field theory, we propose a simple thought experiment based on particles interacting via semiclassical forces. Surprisingly a simple consistency argument with relativistic causality is enough to obtain a fundamental result which, being related to gravitational and electric fields, indirectly tells us something about quantum gravity and quantum field theory.

The result is the following: assuming that a macroscopic mass *m* is prepared in a superposition of two states separated by a distance *d*, then any experiment discriminating the coherent superposition from a classical incoherent mixture requires a minimum time *T* ∝ *md*, proportional to the mass and the separation distance. Analogously for a quantum superposition of a macroscopic charge *q*, such minimum time is proportional to the associated electric dipole *T* ∝ *qd*. In a nutshell, experiments testing macroscopic superpositions are possible in principle, but they need to be slow. For common experiments involving systems below the Planck mass and the Planck charge this limitation is irrelevant, however such time can become very important at macroscopic scales. As an extreme example, if the center of mass of the Earth were in a quantum superposition with a separation distance of one micrometer, according to our result one would need a time equal to the age of the universe in order to distinguish this state from a classical statistical mixture. Clearly this limitation suggests that at sufficiently macroscopic scales quantum mechanics can be safely replaced by classical statistical mechanics without noticing the difference.

The fact that large gravitational or electromagnetic fields can be a limitation for the observation of quantum superpositions is not a new idea. In the past decades, several models of spontaneous localization^11^,^12^,^13^,^14^,^15^ have been proposed which, going beyond the standard theory of quantum mechanics, postulate the existence of a gravity induced collapse at macroscopic scales. Remaining within the domain of standard quantum mechanics, the loss of coherence in interference experiments due to the emission of electromagnetic radiation has been already studied in the literature^16^,^17^. Similarly, the interaction of a massive particle with gravitational waves^18^,^19^,^20^ and the dephasing effect of time dilation on internal degrees of freedom^21^ have been considered as possible origins of quantum decoherence.

For what concerns our thought experiment, a similar setup can be found in the literature where the interference pattern of an electron passing through a double slit is destroyed by a distant measurement of its electric field. This thought experiment can be traced back to Bohr as quoted in^16^, was discussed by Hardy interviewed in^22^ and appears as an exercise in the book by Aharanov and Rohrlich^23^. Recently different experiments involving interacting test particles have been proposed in order to discriminate the quantum nature of the gravitational field from a potentially classical description^24^,^25^, while some limitations that relativistic causality imposes to the possible measurements in quantum field theory have been investigated in^26^.

The original contribution of our work is that, imposing the consistency with relativistic causality, our thought experiment allows the derivation of a fundamental minimum time which is valid for *any* possible experiment involving macroscopic superpositions. In this sense our bounds represent universal limitations having a role analogous to the Heisenberg uncertainty principle in quantum mechanics. For this reason, while our results could be observable in advanced and specific experimental setups^27^,^28^,^29^,^30^,^31^,^32^,^33^,^34^, their main contribution is probably a better understanding of the theory of quantum mechanics at macroscopic scales. For charged particles we propose two different measurements for testing the coherence. The first requires to accelerate the charge, and our bound on the discrimination time is due to the entanglement with the emitted photons. In the second, the bound can be instead ascribed to the presence of the vacuum fluctuations of the electromagnetic field. On the other hand we also find an equivalent bound associated to quantum superposition of large masses. What is the origin of this limitation? The analogy suggests that the validity of our bound could be interpreted as an indirect evidence for the existence of quantum fluctuations of the gravitational field, and of quantum gravitational radiation.

## Thought experiment

**Figure 1:**
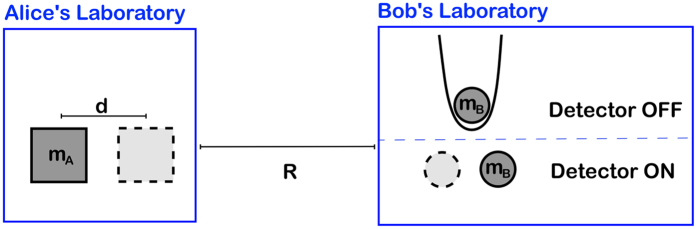
Picture of the thought experiment. Alice prepares a macroscopic mass in a quantum spatial superposition. Bob has at disposal a test mass prepared in the ground state of a narrow harmonic trap. Bob can send one bit of information to Alice by choosing between two alternatives: doing nothing (detector *off*  ) or removing the trap (detector *on*). Once a time *T*_B_ necessary to generate entanglement (if the detector is *on*) has passed, Alice performs a measurement in a time *T*_A_ in order to discriminate the coherent superposition from a classical incoherent mixture. In this way, by knowing whether the detector is *on* or *off*, she gets the information sent by Bob in a time *T*_A_ + *T*_B_. A completely equivalent protocol can be obtained by replacing massive particles with charged particles.

Consider the thought experiment represented in Fig. 1, and described by the following protocol. The protocol can be equivalently applied to quantum superpositions of large masses or large charges.

### Protocol of the thought experiment

1. Alice has at disposal, in her laboratory, a massive/charged particle in a macroscopic superposition of a “left” and a “right” state:


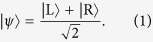


The wave functions of the two states are 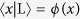 and 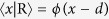, where *d* > 0 is the relative separation of the superposition.

2. Bob is in a laboratory at a distance *R* from Alice and containing a massive/charged test particle prepared in the ground state of a very narrow harmonic trap. Bob freely chooses between two options: doing nothing (detector = *off*  ), or removing the trap (detector = *on*). In the first case the state of test particle remains unchanged while, in the second case, the dynamics is sensitive to the local Newton/Coulomb field generated by Alice’s particle and the global state will eventually become entangled. If the detector is *off*, the initial quantum superposition is preserved, while if the detector is *on* the generation of entanglement eventually destroys the coherence of the reduced state of Alice.

3. Alice performs an arbitrary experiment in her laboratory with the task of discriminating the coherent superposition from a statistical incoherent mixture of the two states 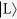 and 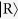. For example, she could make an interference experiment, a measurement of the velocity, or she could measure the gravitational/electromagnetic field (or some spatial average of it) in any point within her laboratory. The specific details of the experiment are irrelevant. Depending on the result of the experiment, Alice deduces the choice of Bob (*i.e.* if the detector was *on* or *off*  ).

Clearly, the previous thought experiment constitutes a communication protocol in which Bob can send information to Alice. Moreover, for a large enough mass *m* or for a large enough charge *q*, the test particle of Bob can become entangled with Alice’s particle in an arbitrarily short time. But then, apparently, Bob can send a message to Alice faster than light violating the fundamental principle of relativistic causality. How can we solve this paradox? Let us make a list of possible solutions:

(a) It is impossible to prepare a macroscopic superposition state or to preserve its coherence because of some unknown intrinsic effect lying outside the theory of quantum mechanics.

(b) Once the superposition is created, the particle is entangled with its own static gravitational/electric field, and Alice’s local state is always mixed. Then she cannot distinguish a coherent superposition from the corresponding incoherent statistical mixture with an experiment inside her laboratory, since the probability distribution of the outcomes of any measurement she can perform does not depend on Bob’s choice. Notice that, if this were the solution, the protocol not only would not allow for superluminal communication, but it would not allow for communication at all.

(c) Alice needs a minimum time to locally discriminate whether the superposition is coherent or not. More quantitatively we have that, if Bob is able to generate entanglement in a time *T*_B_ and if *T*_A_ is the time necessary to Alice for performing her discrimination measurement, then relativistic causality requires


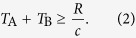


Therefore, whenever entanglement can be generated in a time *T*_B_ ≤ *R*/*c*, we get a non-trivial lower bound on *T*_A_. Here we are neglecting the time necessary to Bob for switching from *off* to *on* the detector, *i.e.* for removing the trap confining the particle. In Appendix H, we justify the validity of this approximation.

Anomalous decoherence effects^11^,^12^,^13^,^14^,^15^ (as *e.g.* the Penrose spontaneous localization model) are important open problems in the foundations of quantum mechanics and cannot be excluded a priori. Up to now however their existence was never experimentally demonstrated and therefore, instead of closing our discussion by directly invoking point a), we try to remain within the framework of quantum mechanics and check if points b) or c) are plausible solutions.

The reader who is familiar with the field of open quantum systems may find the option b) very natural. In standard non-relativistic quantum mechanics, the formation of entanglement between a system and its environment is widely accepted as the origin of any observed form of decoherence. Indeed this approach has also been used to explain the decoherence of moving charged particles, mainly focusing to the double-slit interference experiment^17^. It has been recognized by previous works that in a double-slit experiment there is a limit to the charge of the particle above which photons are emitted due to the acceleration associated to the interference paths^16^,^17^. For large charges then, the particle entangles with the emitted photons and this effect can destroy the interference pattern. The reader can then notice that also in our case the particle needs to be accelerated when it is put in the superposition (1), and if it is charged it will radiate and can become entangled with the emitted photons. Similarly, an accelerated mass generates gravitational radiation and can become entangled with the emitted gravitons. However, in Appendix E we prove that, if the accelerations are slow enough, the resulting quantum state of the electromagnetic field has almost overlap one with the vacuum, and therefore the particle does not get entangled with the emitted photons because no photons at all are emitted. The same argument can be repeated for the gravitational radiation in the linear approximation.

The reader may now think that the particle in the superposition (1) is entangled at least with its static Coulomb electric field. However, as we show in details in Appendix F, the static Coulomb electric field is not a propagating degree of freedom (it has zero frequency) and vanishes in absence of electric charges. In the Coulomb gauge, the Hilbert space associated to the static field is the same Hilbert space of the particle, whose reduced state remains pure. Indeed, the quantum operator associated to the electric field contains explicitly the operator associated to the position of the particle, and then the expectation value of the electric field can be non-vanishing and depends on the state of the particle even if all the propagating modes of radiation are in their vacuum state and there is no entanglement.

In other gauges entanglement can be present. However, contrarily to what usually happens, the presence of entanglement by itself does not prevent Alice to distinguish a coherent superposition from a statistical mixture. Indeed, as we will show later, Alice can exploit an internal degree of freedom of the particle to remove this entanglement with a local operation, and then perform an experiment only on the internal degree of freedom to test the coherence. The operation consists in bringing the right wavepacket of the superposition 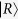 to the left position 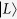, while leaving the left wavepacket 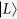 untouched. Independently on the gauge, the particle will then have a definite position and also its own static Coulomb field will be definite. This proves that the protocol allows for communication, and the solution b) is wrong in the sense that if Alice could perform in a sufficiently short time the above local operation, superluminal communication would still be possible.

The reasonable solution to the paradox appears then to be the final option c). Basically, even if the state of the particle is pure and coherent, Alice cannot instantaneously test this fact with a local experiment in her laboratory. Notice that the hypothesis c) is weaker than hypothesis a), and the two can logically coexist. Clearly if a) is valid Alice cannot make any useful experiment because decoherence has already happened. Therefore we conclude that the weaker and most general solution to the paradox is the fundamental limitation exposed in point c). In the last part of this paper, we propose two different measurements and show that they are both consistent with this limitation.

## Minimum discrimination time

In the previous section we argued that relativistic causality requires a fundamental limitation: Alice’s discrimination experiment must be slow. But how slow it has to be? By construction any thought experiment of the class described before gives a lower bound on the discrimination time *T*_A_ whenever *T*_B_ ≤ *R*/*c*. In what follows we are going to optimize over this class of experiments. We anticipate that this approach leads to the following two bounds which constitute the main results of this work.

### Minimum discrimination time for quantum superpositions of large masses

Given a particle of mass *m* prepared in a macroscopic quantum superposition of two states separated by a distance *d*, it is impossible to locally discriminate the coherent superposition from an incoherent mixture in a time (up to a multiplicative numerical constant) less than


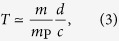


where *m*_p_ is the Planck mass


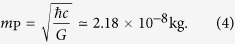


### Minimum discrimination time for quantum superpositions of large charges

Given a particle of charge *q* prepared in a macroscopic quantum superposition of two states separated by a distance *d*, it is impossible to locally discriminate the coherent superposition from an incoherent mixture in a time (up to a multiplicative numerical constant) less than


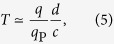


where *q*_p_ is the Planck charge


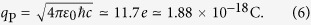


Before giving a derivation of the previous results, we stress that both the bounds (3) and (5) are relevant only for *q* ≥ *q*_*p*_ and *m* ≥ *m*_*p*_. Indeed for systems below the Planck mass/charge, even if the bounds are formally correct, their meaning is trivial since any measurement of the state must at least interact with both parts of the superposition and this process requires at least a time *d*/*c*.

## Dynamics of Bob’s test mass

Let us first focus on the superpositions of massive particles and give a proof of the bound (3) (the proof of (5) is analogous and will be given later). It is easy to check that, for a sufficiently narrow trap (detector = *off*  ) the test mass of Bob is insensitive to the gravitational force of Alice’s particle and remains stable in its ground state (see Appendix A for details). On the contrary, if the trap is removed, the test mass will experience a different force depending on the position of Alice’s particle. The two corresponding Hamiltonians are:


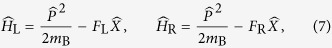


where *m*_B_ is the mass of Bob’s particle, and *F*_L_ and *F*_R_ are the different gravitational forces associated to the “left” and “right” positions of Alice’s particle. Their difference


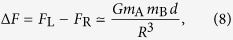


where *m*_A_ is the mass of Alice’s particle, determines the dipole force sensitivity that Bob should be able to detect in order to induce the decoherence of the reduced state possessed by Alice.

Given the initial state of the test mass 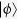, it is easy to check that entanglement can be generated in a time *t* whenever the different time evolutions associated to 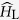 and 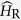 drive the test mass into almost orthogonal states, *i.e.*


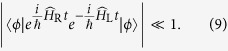


Such time depends on the initial state 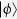 and on the Loschmidt echo operator


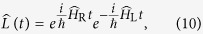


which after two iterations of the Baker-Campbell-Hausdorff formula can be written as


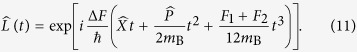


Neglecting the complex phase factor 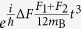, 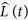 is essentially a phase–space displacement operator of the form 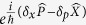, where


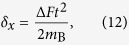



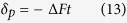


are the shifts in position and momentum, respectively.

Since the initial state 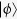 of the test mass is the ground state of a very narrow harmonic trap, it will correspond to a localized Gaussian wave packet which is very noisy in momentum and therefore we may focus only on the position shift (12) and compare it with the position uncertainty Δ*X* of the initial state (see Appendix A for a detailed proof). We can argue that entanglement is generated only after a time *t* = *T*_B_ such that


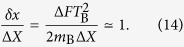


Apparently Bob can generate entanglement arbitrarily quickly by reducing the position uncertainty Δ*X*. However there is a fundamental limit to the localization precision which is set by the Planck length. It is widely accepted that no reasonable experiment can overcome this limit^35^,^36^,^37^:


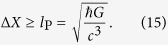


From Eq. (14), substituting Eq. (8) and using the minimum Δ*X* allowed by the constraint (15), we get


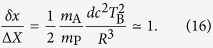


As we have explained in the previous section, relativistic causality implies the inequality (2) involving Alice’s measurement time *T*_A_ and the entanglement time *T*_B_. Such inequality provides a lower bound on *T*_A_ only if *T*_B_ < *R*/*c* while it gives no relevant information for *T*_B_ ≥ *R*/*c*. Therefore we parametrize *R* in terms of *T*_B_ and a dimensionless parameter *η*:


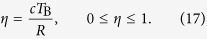


Using this parametrization, from Eq. (16), we get


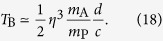


From the causality inequality (2) we have


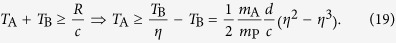


Optimizing over *η* we get


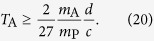


This is, up to a multiplicative numerical constant, the bound given in Eq. (3).

## Dynamics of Bob’s test charge

The calculation in the case in which we have a test charge instead of a test mass is almost identical. The only difference is that Eq. (8) is replaced by the Coulomb counterpart


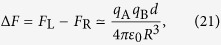


where *q*_A_ and *q*_B_ are the charges of Alice’s and Bob’s particles, respectively, while the localization limit (15) is replaced by Bob’s particle charge radius^38^


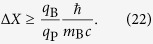


More details on the minimum localization of a macroscopic charge are given in Appendix B. From Eqs (21) and (22), repeating exactly the previous argument one finds


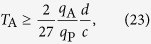


which is, up to a multiplicative numerical constant, the bound given in Eq. (3).

## Minimum time from entanglement with radiation

In the previous section we have proved that relativistic causality requires that any measurement Alice can perform to test the coherence of her superposition must require a minimum time, depending on the mass or charge of her particle. Here we focus on the electromagnetic case, and propose two different measurements to check the coherence of the superposition.

The first is a simplified version of the experiment proposed in refs 33,34. Let Alice’s particle have spin 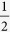, and suppose that her superposition is entangled with the spin, i.e. (1) is replaced by


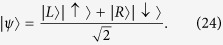


Let now Alice apply a spin-dependent force, that vanishes if the spin is up, while brings the particle from 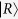 to 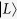 if the spin is down. Notice that, at the end of this operation, the charge has a well-defined position, and also its own static Coulomb electric field is well-defined. In this way, the original macroscopic superposition has been reduced to a microscopic spin superposition, on which an instantaneous discrimination experiment can be performed.

If Bob does not perform the measurement, the final state of Alice’s particle is


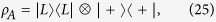


where


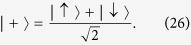


On the contrary, if Bob induces a collapse of the wave-function, the final state is


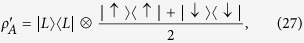


and Alice can test the coherence by measuring the spin.

However, this protocol requires the particle to be accelerated if it has spin down and needs to be moved from 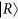 to 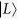. Then it will radiate, and it can entangle with the emitted photons. A semiclassical computation of the emitted radiation can be found in Appendix E. There we show that such radiation is indistinguishable from the vacuum state of the field only if the motion lasts for at least the time required by our previous bound (5).

## Minimum time from quantum vacuum fluctuations

In the previous section we have provided an example of experiment able to test the coherence. The protocol requires to accelerate the charge, and if its duration is too short, the charge radiates and entangles with the emitted photons. The reader could now think that the bound on the time could be beaten with an experiment that does not involve accelerations. An example of such experiment could seem to be a measurement of the canonical momentum of Alice’s particle. In this section, we first show that this measurement is indeed able to test the coherence of the superposition and then we estimate the minimum time necessary to perform it.

The canonical momentum of a charged particle coupled to the electromagnetic field is not gauge invariant, and therefore cannot be directly measured. Alice can instead measure directly the velocity of her particle, that is gauge invariant. However, its relation with the canonical momentum now contains the vector potential. Even if there is no external electromagnetic field, the latter is a quantum-mechanical entity, and is subject to quantum vacuum fluctuations. Then, the fluctuations of the vector potential enter in the relation between velocity and momentum. If Alice is not able to measure the field outside her laboratory, she can measure only the velocity of her particle (see Appendix F for a detailed discussion), and can reconstruct its canonical momentum only if the fluctuations are small. We show that in an instantaneous measurement these fluctuations are actually infinite. However, if Alice measures the average of the velocity over a time *T*, they decrease as 1/*T*^2^, and can be neglected if *T* is large enough. This minimum time is found consistent with the general bound given in Eq. (5).

In order to simplify our formulas, in this section and in the related Appendices we put as in^38^


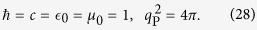


These constants will be put back into the final result.

## The canonical momemtum as a test for coherence

Let us first show that Alice can test the coherence with a measurement of the canonical momentum of her particle.

Let the particle be in the coherent superposition (1) of two identical wave-packets centered in different points, with wave-function


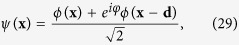


where *φ* is an arbitrary phase.

The probability distribution of the canonical momentum 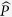 is the modulus square of the Fourier transform of the wave-function:


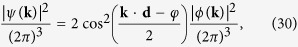


and she can test the coherence of the superposition from the interference pattern in momentum space generated by the cosine. Indeed, an incoherent statistical mixture would be associated to the probability distribution


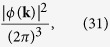


where the cosine squared is replaced by 1/2, its average over the phase *φ*.

Notice from (30) that, in order to be actually able to test the coherence, Alice must measure the canonical momentum with a precision of at least


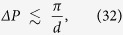


where 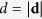. This precision increases with the separation of the wave-packets, e.g. for *d* = 1m, it is 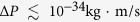.

## Quantum vacuum fluctuations and minimum time

Let now Alice’s particle carry an electric charge *q*. We want to take into account the quantum vacuum fluctuations of the electromagnetic field, so quantum electrodynamics is required. The global Hilbert space is then the tensor product of the Hilbert space of the particle 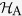 with the Hilbert space of the field 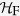. The reader can find in Appendix C the details of the quantization.

The position and canonical momentum operators of Alice’s particle 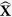 and 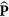 still act in the usual way on the particle Hilbert space alone, so that the argument of the previous sub subsection remains unchanged. The full interacting Hamiltonian of the particle and the electromagnetic field is


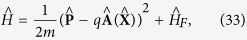


where


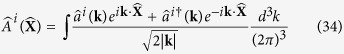


is the vector-potential operator 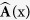 (see Eq. (C5) of the Supplementary Material) with the coordinate **x** replaced with the position operator 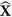, and 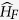 is the free Hamiltonian of the electromagnetic field defined in Eq. (C12) of the Supplementary Material.

Due to the minimal-coupling substitution, the operator associated to the velocity of the particle is


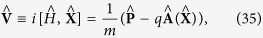


that contains the operator vector-potential, and acts also on the Hilbert space of the field. The canonical momentum can be reconstructed from the velocity with


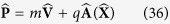


if the second term in the RHS can be neglected. With the help of the commutation relations (see Eq. (C6) of the Supplementary Material), a direct computation of the variance of 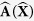 on the vacuum state of the field gives


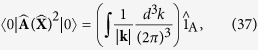


that has a quadratic divergence for 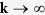 due to the quantum vacuum fluctuations. This divergence can be cured averaging the vector potential over time with a smooth function *φ*(*t*). We must then move to the Heisenberg picture, where operators explicitly depend on time, and we define it to coincide with the Schrödinger picture at *t* = 0, the time at which Alice measures the velocity. Since the divergence in (37) does not depend neither on the mass nor on the charge of Alice’s particle and is proportional to the identity operator on the particle Hilbert space 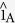, it has nothing to do with the interaction of the particle with the field. Then the leading contribution to the result can be computed evolving the field with the free Hamiltonian 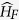 only, i.e. with


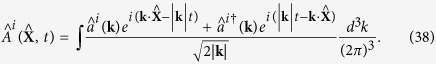


Defining the time-averaged vector potential as


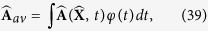


its variance over the vacuum state of the field is now


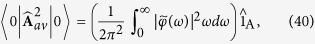


where


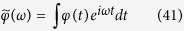


is the Fourier transform of *φ*(*t*). Taking as *φ*(*t*) a normalized Gaussian function of width *T* centered at *t* = 0:


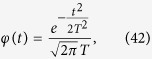


we get as promised a finite result proportional to 1/*T*^2^:


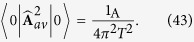


Then, if Alice estimates one component of the canonical momentum (say the one along the *x* axis) with the time average of the velocity taken with the function *φ*(*t*), she commits an error of the order of


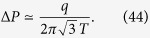


Comparing (44) with the required precision to test the coherence (32), the minimum time required is


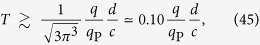


in agreement with the bound (23) imposed by relativistic causality alone.

## Discussion

In this work we have studied the limitations that the gravitational and electric fields produced by a macroscopic particle impose on quantum superposition experiments. We found that, in order to avoid a contradiction between quantum mechanics and relativistic causality, a minimum time is necessary in order to discriminate a coherent superposition from an incoherent statistical mixture. This discrimination time is proportional to the separation distance of the superposition and to the mass (or charge) of the particle.

In the same way as the Heisenberg uncertainty principle inspired the development of a complete theory of quantum mechanics, our fundamental and quantitative bounds on the discrimination time can be useful for the development of current and future theories of quantum gravity. Moreover, despite an experimental observation of our results clashes with the difficulty of preparing superpositions of masses above the Planck scale, the current technological progress on highly massive quantum optomechanical and electromechanical systems provides a promising context^27^,^28^,^29^,^30^,^31^,^32^,^33^,^34^ for testing our predictions.

## Additional Information

**How to cite this article**: Mari, A. *et al.* Experiments testing macroscopic quantum superpositions must be slow. *Sci. Rep.* **6**, 22777; doi: 10.1038/srep22777 (2016).

## Supplementary Material

Supplementary Information
