# Supplementary material for: Experiments testing macroscopic quantum superpositions must be slow
Source: Sci Rep. 2016 Mar 9;6:22777. doi: 10.1038/srep22777 (PMC4784303; doi:10.1038/srep22777)
Supplement: Supplementary Information [file srep22777-s1.pdf]

Supplementary information
Experiments testing macroscopic quantum superpositions must be slow

Andrea Mari,[1] Giacomo De Palma,[1,2] and Vittorio Giovannetti[1]

[1]*NEST, Scuola Normale Superiore and Istituto Nanoscienze-CNR, I-56126 Pisa, Italy*
[2]*INFN, Pisa, Italy*

Appendix A: Strength of the trap

In this Appendix we show that for Bob a measurement of the position is always better than a measurement of the momentum, *i.e.* it allows to distinguish the force difference ΔF in a shorter time.

Let ω be the frequency of the harmonic trap. The spatial width of its ground state is given by

$$\Delta X^2 \simeq \frac{\hbar}{m_{\rm B}\,\omega} \; . \tag{A1}$$

This ground state is insensible to the force difference ΔF iff the displacement that it generates is less than ΔX, i.e.

$$\frac{\Delta F}{m_{\rm B}\,\omega^2} \lesssim \Delta X \; . \tag{A2}$$

Eliminating ω with (A1), the inequality (A2) becomes hence

$$\Delta X^3 \lesssim \frac{\hbar^2}{m_{\rm B}\,\Delta F} \; , \tag{A3}$$

which is the condition we have to enforce to ensure that Bob's detector is ineffective when switched off. Suppose then that, after switching on the detector, Bob tries to distinguish the two states of Alice by a measurement of P: accordingly the momentum spread ΔP of his initial state must be lower than the displacement in momentum $|\delta_p| = \Delta F\, t$. Recalling that Heisenberg's uncertainty principle $\Delta X\,\Delta P \geq \hbar$ is saturated by a Gaussian pure state, the minimum time after which Bob can distinguish is

$$T'_{\rm B} = \frac{\hbar}{\Delta F\,\Delta X} \; . \tag{A4}$$

On the other hand, from the ratio considered in the main text

$$\frac{\delta x}{\Delta X} = \frac{\Delta F\, T_{\rm B}^2}{2 m_{\rm B} \Delta X} \simeq 1, \tag{A5}$$

the minimum discrimination time with a measurement of x is

$$T_{\rm B} = \sqrt{\frac{m_{\rm B}\,\Delta X}{\Delta F}} \; . \tag{A6}$$

The reader can check that (A3) implies $T_{\rm B} \leq T'_{\rm B}$, i.e. if the trap is strong enough to be insensible to the force difference, for Bob it is always better to measure the position of his particle rather than its momentum.

Appendix B: Maximum localization of a charge

In this Appendix we prove that the minimum width over which a charge q greater than the Planck charge q_P can be localized is its charge radius $q\hbar/(q_\mathrm{P}mc)$ [1].

Let us suppose to use a harmonic trap of frequency ω to localize the charge. One could think that in principle, with a strong enough trap, the charge can be arbitrarily localized. However, from the Larmor formula [2] we know that a classical particle with charge q following a harmonic motion of frequency ω and width ΔX loses into electromagnetic radiation a power

$$\frac{dE}{dt} \simeq \frac{q^2\,\omega^4\,\Delta X^2}{\epsilon_0\,c^3}\ . \tag{B1}$$

In the quantum case, the charge radiates until it gets to the ground state of the trap, where it cannot radiate anymore since there are no other states with a lower energy to go. However, if the trap is very strong, its ground state is very localized, and therefore has a great uncertainty in velocity. Since any moving charge generates a magnetic field, this velocity uncertainty generates a large uncertainty in the magnetic field, resulting in a large entanglement between the state of the particle and the state of the field. Qualitatively, this happens when the energy classically radiated in a period becomes greater than $\hbar\omega$, the energy of the first excited state. Combining (B1) with (A1), this happens exactly when the localization ΔX becomes smaller that the charge radius:

$$\Delta X \lesssim \frac{q}{q_\mathrm{P}}\,\frac{\hbar}{mc}\ . \tag{B2}$$

Then, if we want the reduced state of the particle to remain pure, we can localize it only up to the limit in (B2).

Appendix C: Quantization of the electromagnetic field

We recall here the basics of the quantization of the electromagnetic field. More details can be found in Ref. [3].

We denote with $\hat{O}$ an operator in the Schrödinger picture, and with $\hat{O}(t)$ its Heisenberg-picture counterpart. The two pictures are defined to coincide for $t=0$, i.e. $\hat{O}(0)=\hat{O}$. We recall that in the Heisenberg picture the operators are evolved with the full interacting Hamiltonian.

An Hamiltonian formulation of electrodynamics requires the introduction of the scalar and vector potentials V and $\mathbf{A}$. It is convenient to Fourier-transform with respect to $\mathbf{x}$. The potentials are related to the electric and magnetic fields by

$$\hat{\mathbf{E}}(\mathbf{k},t) \;=\; -i\mathbf{k}\,\hat{V}(\mathbf{k},t) - \frac{\partial}{\partial t}\hat{\mathbf{A}}(\mathbf{k},t) \tag{C1}$$

$$\hat{\mathbf{B}}(\mathbf{k},t) \;=\; i\mathbf{k}\times\hat{\mathbf{A}}(\mathbf{k},t)\ . \tag{C2}$$

We choose the Coulomb gauge, in which the divergence of the vector potential is set to zero at the operator level:

$$\mathbf{k}\cdot\hat{\mathbf{A}}(\mathbf{k},t) = 0\ . \tag{C3}$$

It is now convenient to define the ladder operators

$$\hat{a}^i(\mathbf{k},t) \equiv \sqrt{\frac{|\mathbf{k}|}{2}}\,\hat{A}^i(\mathbf{k},t) + \frac{i}{\sqrt{2|\mathbf{k}|}}\,\frac{\partial}{\partial t}\hat{A}^i(\mathbf{k},t)\ , \tag{C4}$$

satisfying the constraint $k_i\,\hat{a}^i(\mathbf{k},t) = 0$ as a consequence of (C3). The definition in (C4) can be inverted:

$$\hat{A}^i(\mathbf{x},t) = \int \frac{\hat{a}^i(\mathbf{k},t)\, e^{i\mathbf{k}\cdot\mathbf{x}} + \hat{a}^{i\dagger}(\mathbf{k},t)\, e^{-i\mathbf{k}\cdot\mathbf{x}}}{\sqrt{2|\mathbf{k}|}}\, \frac{d^3k}{(2\pi)^3}\ . \tag{C5}$$

The ladder operators satisfy the equal-time canonical commutation relations

$$\left[\hat{a}^i(\mathbf{k},t),\ \hat{a}^{j\dagger}(\mathbf{q},t)\right] = \Pi^{ij}(\mathbf{k})\,(2\pi)^3\delta^3(\mathbf{k}-\mathbf{q}) \tag{C6}$$

$$\left[\hat{a}^i(\mathbf{k},t),\ \hat{a}^{j}(\mathbf{q},t)\right] = \left[\hat{a}^{i\dagger}(\mathbf{k},t),\ \hat{a}^{j\dagger}(\mathbf{q},t)\right] = 0\ , \tag{C7}$$

where $\Pi(\mathbf{k})$ is the projector onto the subspace orthogonal to $\mathbf{k}$:

$$\Pi^{ij}(\mathbf{k}) = \delta^{ij} - \frac{k^i\,k^j}{\mathbf{k}^2}\ . \tag{C8}$$

The vacuum state of the field $|0\rangle$ is defined as the state annihilated by all the Schrödinger-picture annihilation operators:

$$\hat{a}^i(\mathbf{k})|0\rangle = 0 \qquad \forall\,\mathbf{k}\in\mathbb{R}^3\ ,\quad i = 1,\,2,\,3\ ,\qquad |0\rangle \in \mathcal{H}_F\ . \tag{C9}$$

Besides, the $\hat{a}^i(\mathbf{k})$ together with their hermitian conjugates $\hat{a}^{i\dagger}(\mathbf{k})$ generate the whole observable algebra of $\mathcal{H}_F$.

Maxwell's equations determine the time evolution of the ladder operators:

$$\frac{\partial}{\partial t}\hat{a}^i(\mathbf{k},t) + i|\mathbf{k}|\,\hat{a}^i(\mathbf{k},t) = \frac{i\,\Pi^i{}_j(\mathbf{k})}{\sqrt{2|\mathbf{k}|}}\hat{J}^i(\mathbf{k},t)\ , \tag{C10}$$

where $\hat{\mathbf{J}}$ is the operator associated to the current density of the quantum system interacting with the electromagnetic field. Eq. (C10) is easily integrated:

$$\hat{a}^i(\mathbf{k},t) = $$
$$= e^{-i|\mathbf{k}|t}\left(\hat{a}^i(\mathbf{k}) + \frac{i\,\Pi^i{}_j(\mathbf{k})}{\sqrt{2|\mathbf{k}|}}\int_0^t e^{i|\mathbf{k}|t'}\hat{J}^i(\mathbf{k},t')\,dt'\right)\ , \tag{C11}$$

where we have imposed the Heisenberg and Schrödinger pictures to coincide at $t = 0$, i.e. $\hat{a}^i(\mathbf{k},0) = \hat{a}^i(\mathbf{k})$.

In the free case, i.e. when the current vanishes at the operator level ($\hat{\mathbf{J}}(\mathbf{k},t) = 0$), the relation between the two pictures is given by the free Hamiltonian

$$\hat{H}_F \equiv \int |\mathbf{k}|\ \hat{a}_i^\dagger(\mathbf{k})\ \hat{a}^i(\mathbf{k})\ \frac{d^3k}{(2\pi)^3}\ , \tag{C12}$$

i.e.

$$\hat{a}^i(\mathbf{k},t) = e^{-i|\mathbf{k}|t}\hat{a}^i(\mathbf{k}) = e^{i\hat{H}_F t}\ \hat{a}^i(\mathbf{k})\ e^{-i\hat{H}_F t}\ . \tag{C13}$$

Appendix D: Coherent states

We introduce now the set of coherent states of the electromagnetic field. Our formalism is analogue to the one of Ref. [4], where the reader is referred for further details.

For any function $f : \mathbb{R}^3 \to \mathbb{C}^3$ subject to the constraint

$$k_i\, f^i(\mathbf{k}) = 0 \qquad \forall\, \mathbf{k} \in \mathbb{R}^3 \ , \tag{D1}$$

define the unitary displacement operator

$$\hat{D}[f] \equiv \exp\left(\int \left(f_i(\mathbf{k})\, \hat{a}^{i\dagger}(\mathbf{k}) - f_i^*(\mathbf{k})\, \hat{a}^i(\mathbf{k}) \right) \frac{d^3k}{(2\pi)^3} \right) \ , \tag{D2}$$

$$\hat{D}^\dagger[f] = \hat{D}[-f] \ , \tag{D3}$$

acting on the ladder operators as

$$\hat{D}^\dagger[f]\ \hat{a}^i(\mathbf{k})\ \hat{D}[f] = \hat{a}^i(\mathbf{k}) + f^i(\mathbf{k}) \ . \tag{D4}$$

Their composition rule is

$$\begin{aligned} &\hat{D}[f]\ \hat{D}[g] = \hat{D}[f+g] \times \\ &\times \exp\left(\frac{1}{2} \int \left(f^i(\mathbf{k})\, g_i^*(\mathbf{k}) - f_i^*(\mathbf{k})\, g^i(\mathbf{k}) \right) \frac{d^3k}{(2\pi)^3} \right) \ . \end{aligned} \tag{D5}$$

We can now define the coherent states with a displacement operator acting on the vacuum state of the field:

$$|f\rangle \equiv \hat{D}[f]|0\rangle \in \mathcal{H}_F \ , \tag{D6}$$

that are eigenstates of the annihilation operators:

$$\hat{a}^i(\mathbf{k})|f\rangle = f^i(\mathbf{k})|f\rangle \ . \tag{D7}$$

Their overlap is

$$|\langle f|g\rangle|^2 = \exp\left(-\int |f(\mathbf{k}) - g(\mathbf{k})|^2 \frac{d^3k}{(2\pi)^3} \right) \ . \tag{D8}$$

Appendix E: Radiation emitted by Alice's particle

In this Appendix we consider a classical charged particle following an accelerated trajectory and coupled to the quantum electromagnetic field. We show that the radiation classically emitted by the particle induces on the quantum field a displacement operator, and if the initial state is the vacuum, it is brought to a coherent state (see Appendix D for the definition). It turns out that, if the accelerations of the particle are smooth enough, this coherent state has overlap almost one with the vacuum, i.e. it is not distinguishable from it. Then, for a slow motion the particle does not radiate photons at all, and therefore it does not get entangled with the field.

Let $\mathbf{J}$ be the classical current density associated to the trajectory of the particle. Looking at the time evolution equation for the ladder operators (C11), and recalling (D4) and (C13), it is easy to show that such evolution is provided by a displacement operator, i.e.

$$\hat{a}^i(\mathbf{k}, t) = \hat{D}^\dagger[f]\ e^{i\hat{H}_F t}\ \hat{a}^i(\mathbf{k})\ e^{-i\hat{H}_F t}\ \hat{D}[f] \ , \tag{E1}$$

where

$$f^i(\mathbf{k}) = \frac{i\, \Pi^i{}_j(\mathbf{k})}{\sqrt{2|\mathbf{k}|}} \int_0^t e^{i|\mathbf{k}|t'}\, J^i(\mathbf{k}, t')\, dt' \ . \tag{E2}$$

Since the ladder operators generate the whole observable algebra of $\mathcal{H}_F$, if the fields starts in the vacuum, its time-evolved state is the coherent state $e^{-i\hat{H}_F t}|f\rangle$. Its overlap with the vacuum can be computed with (D8):

$$|\langle 0|f\rangle|^2 = \exp\left(-\int |f(\mathbf{k})|^2 \frac{d^3k}{(2\pi)^3}\right) . \tag{E3}$$

We now consider a point particle carrying charge q that starts in $\mathbf{x} = \mathbf{0}$ at $t = 0$, and in a time t_0 is brought to the position $\mathbf{x} = \mathbf{d}$ with a trajectory described by $\mathbf{x}(t)$. The current density is then

$$\mathbf{J}(\mathbf{k}, t) = q\ \mathbf{v}(t)\ e^{-i\mathbf{k}\cdot\mathbf{x}(t)} , \tag{E4}$$

where $\mathbf{v}(t) \equiv \frac{d}{dt}\mathbf{x}(t)$ is the particle velocity. For wavelengths large with respect to the extension of the motion, i.e. for

$$|\mathbf{k}| \ll \frac{1}{d} , \tag{E5}$$

the phase factor in (E4) can be discarded, getting

$$\mathbf{J}(\mathbf{k}, t) \simeq q\ \mathbf{v}(t) . \tag{E6}$$

We want to look at the state of the field after the particle has reached the new position $\mathbf{x} = \mathbf{d}$, i.e. for $t > t_0$. Since the velocity $\mathbf{v}(t)$ vanishes for $t \le 0$ and $t \ge t_0$, the displacement of (E2) becomes

$$f^i(\mathbf{k}) = \frac{i\,q}{\sqrt{2|\mathbf{k}|}}\ \Pi^i{}_j(\mathbf{k})\ v^j(\omega = |\mathbf{k}|) , \tag{E7}$$

where

$$\mathbf{v}(\omega) = \int_{-\infty}^{\infty} \mathbf{v}(t)\ e^{i\omega t}\ dt \tag{E8}$$

is the Fourier transform of the velocity. Putting (E7) into (E3), the overlap becomes

$$|\langle 0|f\rangle|^2 = \exp\left(-\frac{q^2}{6\pi^2}\int_0^{\infty} |\mathbf{v}(\omega)|^2\ \omega\ d\omega\right) . \tag{E9}$$

For simplicity we consider a one-dimensional motion, and we put the x axis in the direction of $\mathbf{d}$. As an example, we take

$$x(t) = d\ \sin^2\left(\frac{\pi}{2}\frac{t}{t_0}\right) \qquad \text{for } 0 \le t \le t_0 , \tag{E10}$$

satisfying the conditions

$$x(0) = 0 , \qquad x(t_0) = d , \qquad v(0) = v(t_0) = 0 . \tag{E11}$$

The Fourier transform of the velocity is

$$v(\omega) = e^{\frac{i\omega t_0}{2}}\ \frac{d\cos\frac{\omega t_0}{2}}{1 - \frac{\omega^2 t_0^2}{\pi^2}} , \tag{E12}$$

and the overlap

$$\begin{aligned} |\langle 0|f\rangle|^2 &= \exp\left(-\pi\,\frac{\pi\mathrm{Si}(\pi)-2}{6}\,\frac{q^2}{q_{\mathrm{P}}^2}\,\frac{d^2}{c^2\,t_0^2}\right) \\ &\simeq \exp\left(-2\,\frac{q^2}{q_{\mathrm{P}}^2}\,\frac{d^2}{c^2\,t_0^2}\right)\,, \end{aligned} \tag{E13}$$

where $\mathrm{Si}(x)$ is the sine integral function

$$\mathrm{Si}(x) \equiv \int_0^x \frac{\sin y}{y} dy\,. \tag{E14}$$

Looking at (E12), the dominant contribution to the integral in (E9) comes from the region $\omega\, t_0 \lessapprox 1$. The approximation in (E5) is then valid iff $d \ll c\, t_0$, i.e. if the motion is not relativistic.

The final result (E13) tells us that for a fixed distance d, no photons are radiated if the motion lasts for at least

$$t_0 \gtrsim \sqrt{2}\,\frac{q}{q_{\mathrm{P}}}\,\frac{d}{c}\,. \tag{E15}$$

Then, Alice can always create the coherent superposition used in the thought experiment without entangling her particle with the emitted photons provided she has enough time to do it. Besides, if Alice wants to perform the particular spin-dependent measurement described in the main text, she needs at least a time (E15) to bring the state $|R\rangle$ back to $|L\rangle$ if she does not want to entangle with the emitted photons.

Appendix F: Absence of entanglement with the static electric field

In this Appendix we explain in detail why when Alice's charged particle is in the quantum superposition considered in the main text, despite it generates a static electric field that depends on its position, it is still not entangled with the field, and the global state is a product with the field part in the vacuum.

The first Maxwell's equation reads

$$\mathbf{k}^2 \hat{V}(\mathbf{k}) = \hat{\rho}(\mathbf{k})\,, \tag{F1}$$

and completely determines the electric potential operator $\hat{V}$ in terms of the charge density operator $\hat{\rho}$:

$$\hat{V}(\mathbf{k}) = \frac{1}{\mathbf{k}^2}\,\hat{\rho}(\mathbf{k})\,. \tag{F2}$$

Putting together (F2) and (C1), the electric field is given by

$$\hat{\mathbf{E}}(\mathbf{k}) = -\frac{i\mathbf{k}}{\mathbf{k}^2}\,\hat{\rho}(\mathbf{k}) - \frac{\partial}{\partial t}\hat{\mathbf{A}}(\mathbf{k})\,. \tag{F3}$$

Then, the longitudinal (i.e. proportional to $\mathbf{k}$) component of the electric field operator is determined by the charge-density operator, and acts on the Hilbert space of the particle alone. Therefore, even if the field is in its vacuum state (C9), the expectation value of the electric field is the static Coulomb electric field generated by the expectation value of the charge density, and hence depends on the particle wave-function. This means that the state of the field alone does not contain all the

information on the electric field, since its longitudinal component is encoded into the state of the particle.

Seen from a different perspective, the longitudinal component of the electric field is not a dynamical propagating degree of freedom, since it vanishes in absence of external charges and is completely determined by them, so there is no Hilbert space associated to it. The Hilbert space of the field contains only the degrees of freedom associated to the electromagnetic radiation, i.e. the magnetic field and the transverse (orthogonal to $\mathbf{k}$) component of the electric field. Then in a product state with the field part in the vacuum, only these components are in the vacuum mode, while there can be a static electric field depending on the state of the particle.

A final remark should be made about the choice of the gauge. Strictly speaking, the concept of particle-field entanglement is a gauge dependent concept and the previous discussion about the absence of entanglement between a static charge and the electromagnetic field is valid only in the Coulomb gauge. However, despite the initial state we consider would look entangled in a different gauge, obviously the estimation of the minimum discrimination time would not change.

Appendix G: Locality

In this Appendix we explain in detail why Alice can measure only the velocity of her particle, and not its canonical momentum, if she is constrained to remain in her laboratory, which has the size of the support of the wave-function of the particle.

The wave-function $\psi(\mathbf{x},t)$ of a particle carrying electric charge q coupled to an electromagnetic field is invariant under the joint gauge transformation [3]

$$\psi'(\mathbf{x},t) = e^{iq\Lambda(\mathbf{x},t)}\,\psi(\mathbf{x},t) \tag{G1}$$

$$\mathbf{A}'(\mathbf{x},t) = \mathbf{A}(\mathbf{x},t) + \nabla\Lambda(\mathbf{x},t) \tag{G2}$$

$$V'(\mathbf{x},t) = V(\mathbf{x},t) - \frac{\partial}{\partial t}\Lambda(\mathbf{x},t)\ . \tag{G3}$$

The canonical momentum $\hat{\mathbf{P}} = -i\nabla$ is not gauge invariant, but transforms in the Heisenberg picture as

$$\hat{\mathbf{P}}'(t) = \hat{\mathbf{P}}(t) + q\nabla\Lambda\left(\hat{\mathbf{X}}(t),t\right)\ , \tag{G4}$$

and therefore Alice cannot measure it directly. The reader can easily check using (G2) and (G4) that the velocity operator given in the main text is gauge invariant, as it has to be. Alice can then measure directly the velocity, and reconstruct from it the canonical momentum. However, the relation between them

$$\hat{\mathbf{P}} = m\,\hat{\mathbf{V}} + q\,\hat{\mathbf{A}}\left(\hat{\mathbf{X}}\right) \tag{G5}$$

contains the vector potential, that from (G2) is not gauge invariant, and cannot be directly measured. In the Coulomb gauge, it is possible to invert (C2) and express the vector potential in terms of the magnetic field, that is gauge invariant and can be actually measured by Alice:

$$\hat{\mathbf{A}}(\mathbf{x}) = \frac{1}{4\pi}\int \frac{\nabla\times\hat{\mathbf{B}}(\mathbf{y})}{|\mathbf{x}-\mathbf{y}|}\,d^3y\ . \tag{G6}$$

Putting together (G6) and (G5), we get

$$\hat{\mathbf{P}} = m\,\hat{\mathbf{V}} + \frac{q}{4\pi}\int \frac{\nabla\times\hat{\mathbf{B}}(\mathbf{y})}{\left|\hat{\mathbf{X}}-\mathbf{y}\right|}\,d^3y\ . \tag{G7}$$

However, reconstructing the canonical momentum from the velocity with (G7) requires Alice to measure the magnetic field in the whole space. Even if she can allow for some error in the reconstruction, the region in which she has to measure the field increases with the charge q, and can extend well outside the support of the wave-function.

Appendix H: Detector switching time

In this work we neglected the time T_D that Bob needs in order to remove the trapping potential and in this Appendix we check the validity of this approximation.

The switching time could be due to many specific technical difficulties but, in principle, the only unavoidable limitation is imposed by relativistic causality. If the linear size of the trap is L, then Bob cannot remove the trap instantaneously since he needs at least a time L/c to induce any change in the apparatus. Moreover the size of the trap cannot be arbitrarily small but should be at least larger than the position uncertainty of the test mass, *i.e.* $L \geq \Delta X$. Thus, a rough estimate of the minimum switching time is given up to numerical factors by

$$T_D \simeq \frac{\Delta X}{c}. \tag{H1}$$

If we want to take into account also this finite response time of the experimental apparatus, the causality equation (2) of the main text should be changed into

$$T_A + T_B + T_D \geq \frac{R}{c}, \tag{H2}$$

where T_A is Alice measurement time and T_B is the entanglement generation time. We remind that the lower bounds for the measurement time that we obtained in this work (for $T_D = 0$) are:

$$T_A \geq \frac{q\,d}{q_{\rm P} c}, \quad q > q_{\rm P} \qquad | \qquad T_A \geq \frac{m\,d}{m_{\rm P} c}, \quad m > m_{\rm P}, \tag{H3}$$

as given by Eq.s (3) and (5) of the main text and corresponding to a quantum superposition of a charge q and mass m respectively. For a finite switching time T_D, from (H1) and (H2) we get the weaker bounds

$$T_A \geq \frac{q\,d}{q_{\rm P} c} - \frac{\Delta X}{c}, \quad q > q_{\rm P}, \qquad | \qquad T_A \geq \frac{m\,d}{m_{\rm P} c} - \frac{\Delta X}{c}, \quad m > m_{\rm P}. \tag{H4}$$

However we remind that in the derivation of the optimal detection experiment we deduced that the trap should be as narrow as possible in order to minimize ΔX (ideally down to the charge radius or to the Planck length). In this regime we always have $d \gg \Delta X$ and the correction terms appearing in Eq.s (H4) are negligible.

---

[1] Weinberg, S. in *The Quantum Theory of Fields*, Vol. I, (Cambridge University Press, 1995).
[2] Jackson, J. D. in *Classical Electrodynamics 3rd edn.*, (Wiley, 1999).
[3] Cohen-Tannoudji, C., Dupont-Roc, J. & Grynberg, G., *Photons and Atoms: Introduction to Quantum Electrodynamics*, (Wiley, 1997).
[4] Barnett, S. M., & Radmore, P. M., *Methods in Theoretical Quantum Optics*, (OUP, 1997).
